# Integrated Opto–Biomechatronics For Single Muscle Fibre Structure‐Function Assessment: The *MyoRobot 3.0*

**DOI:** 10.1002/advs.75731

**Published:** 2026-06-27

**Authors:** Michael Haug, Moritz Hellmann, Larysa Kovbasyuk, Maria Eleni Vazakidou, Milena Theis, Oliver Friedrich

**Affiliations:** ^1^ Institute of Medical Biotechnology, Department of Chemical and Biological Engineering Friedrich‐Alexander‐University Erlangen‐Nürnberg Erlangen Bavaria Germany

**Keywords:** calcium‐dependent elasticity, diameter tracking, fibre‐wide imaging, muscle biomechanics, passive stiffness modulation, stress underestimation

## Abstract

Accurate assessment of biomechanical properties in single muscle fibres requires integrated structural and functional analysis. Conventional approaches assume constant fibre geometry during passive stretch and often use continuous stress–strain and stepwise stretch‐jump protocols interchangeably, despite their differing mechanical implications. We introduce the *MyoRobot 3.0*, a next‐generation biomechatronics platform that integrates automated force measurements with fibre‐wide optical imaging. By synchronising a motorized optical system with controlled stretch velocities, the device maintains a constant field of view during elongation, enabling continuous diameter tracking along the same fibre segment. Using this approach, we quantified diameter thinning during axial stretch and compared slow continuous stress–strain recordings with rapid stepwise stretch‐jumps under varying calcium conditions and with or without cross‐bridge inhibition. Fibre diameter decreased by ∼13–15% at 40% strain, resulting in ∼27% cross‐sectional area loss and systematic underestimation of restoration stress when uncorrected. Continuous stress–strain protocols revealed calcium‐dependent increases in passive stiffness, whereas stepwise stretch‐jumps did not, indicating that the apparent calcium sensitivity partially reflects viscous or time‐dependent contributions. The *MyoRobot 3.0* corrects major structural sources of error and enables protocol‐resolved biomechanical analysis, improving interpretation of passive muscle stiffness and its molecular determinants.

## Graphical abstract

We present the MyoRobot 3.0, an advanced platform that combines high‐resolution imaging with automated mechanical testing to improve the accuracy of single muscle fibre analysis. The system tracks fibre diameter changes during stretch, preventing major stress miscalculations. It also reveals how experimental protocols influence stiffness measurements, refining interpretations of muscle mechanics.

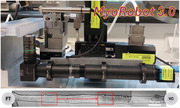

## 1 Introduction

In skeletal muscle physiology, the tight interplay between structure and function is fundamental. Structural characteristics such as sarcomere alignment and fibre geometry directly influence muscle performance [1, 2, 3], while mechanical loading, in turn, modulates structural integrity [4, 5, 6]. Thus, to fully understand muscle performance–whether in health, pathology, or therapeutic interventions, requires simultaneous structural and functional assessments [2, 7, 8, 9]. While several established biomechatronics systems allow the functional analysis of contractile behavior on the cellular level, many of these platforms fall short when it comes to capturing dynamic structural information alongside force measurements [10, 11]. In this context, biomechatronics platforms for muscle fibre analysis play a central role in both basic and translational research. These systems enable the quantification of muscle performance across morphological scales–from whole muscle preparations down to single fibres [8]–providing controlled access to contractile and passive biomechanical properties [12, 13, 14]. At the single‐fibre level in particular, they allow researchers to dissect the sequence of events underlying muscle contraction and to identify dysfunctions within this cascade, such as altered cross‐bridge kinetics or impaired calcium handling [15, 16]. Moreover, such platforms are essential for separating the contributions of structural components, including extracellular matrix remodeling (e.g., fibrosis) and cytoskeletal rearrangements, to passive stiffness in health and disease [17, 18]. As a result, they serve as important tools for understanding muscular disorders, dystrophies, age‐related muscle decline, and for evaluating therapeutic interventions.

**FIGURE 1:**
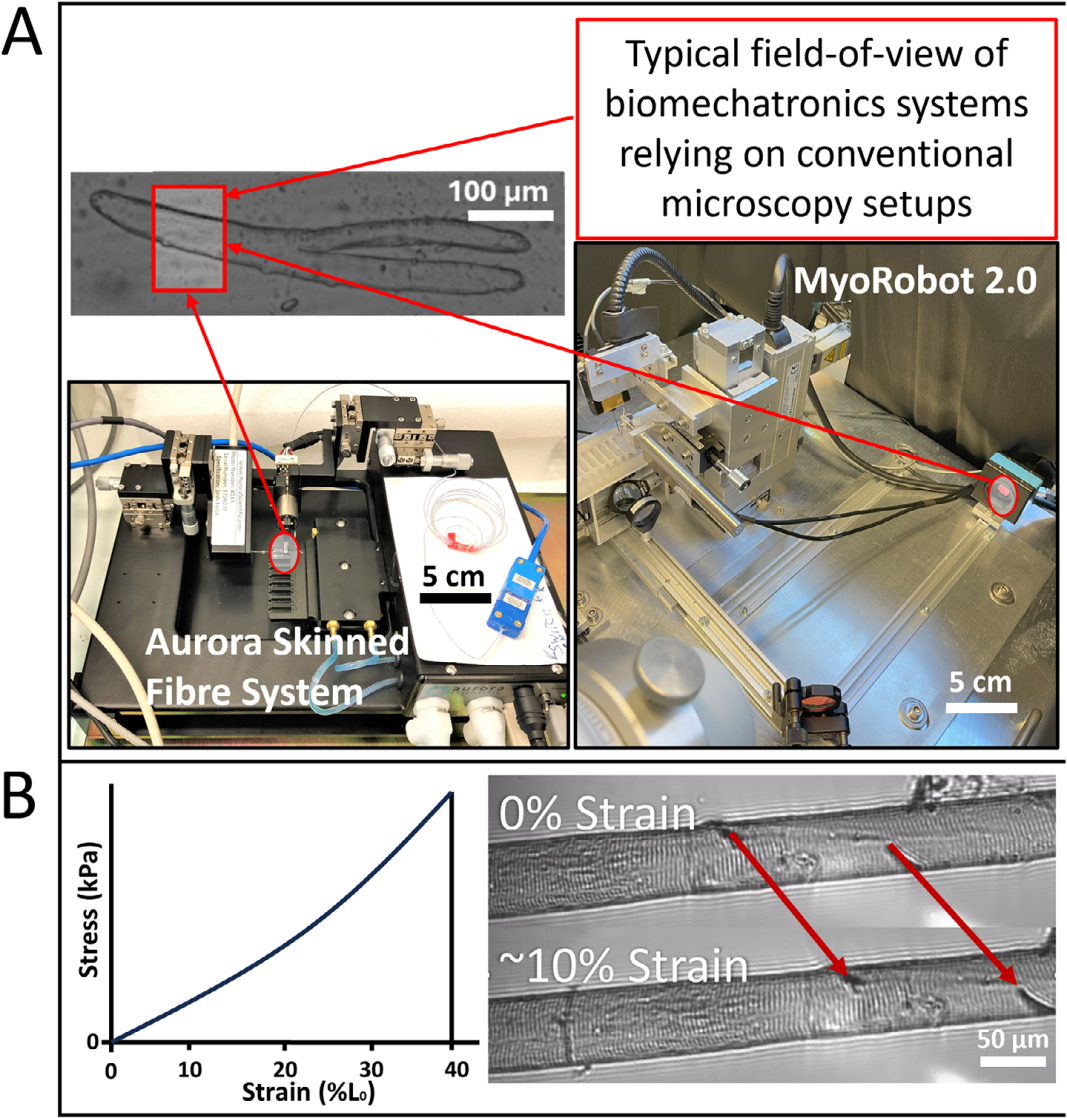
Drawbacks of a limited and static FOV in current biomechatronics systems. (A) a typical FOV with sufficient magnification to detect sarcomeres allows to image a ∼200 μm segment of a single muscle fibre (often less). This area is coarsely highlighted in an image of an entire dystrophic mdx single fibre taken at lower magnification. The loss of structural information from a limited FOV is imminent. (B) particularly in stretch protocols, such as stress–strain recordings sketched here, the muscle fibre shifts out of the FOV. This is highlighted by the red arrows indicating a shift of some marked structures on the fibre over 10% strain.

A critical limitation in conventional muscle biomechatronics setups (either microscope mounted or with integrated optics) is their limited optical field of view (FOV) at higher magnifications (≥20×) needed to visualize the sarcomere lattice (resolution <2μm required). In a typical setup, only a small segment of the fibre is visible during biomechanical recordings (Figure 1A), which can lead to misinterpretation if that segment is not representative of the fibre as a whole. This becomes even more challenging during experiments involving stretch protocols, where the initially imaged section will move and eventually shift out of FOV as the fibre lengthens (Figure 1B). While this can be theoretically prevented in microscope‐mounted biomechanics systems, there is usually no software feedback between the length controller of the system and the microscope's stage. In addition, muscle fibres are not structurally uniform: sarcomere lengths (SL) vary along the longitudinal axis [19, 20] and in vivo [21, 22, 23], and fibre diameter can fluctuate significantly across its length [12]. Consequently, interpreting data based on a fixed, local image can contribute to the already considerable biological and metrological variability in muscle fibre biomechanics and impede data robustness [24].

In an ideal scenario, we would obtain structural information along the entire length of the muscle fibre to complement functional measurements and provide a more accurate basis for calculating biomechanical parameters such as stress (especially under ongoing and varying strain). Achieving this goal, however, presents a technical challenge: even a typical murine muscle fibre (e.g., from an *M. Soleus* or *M. Extensor Digitorum Longus*) can easily exceed 2 mm in length, far beyond the FOV of conventional high resolution microscope objectives. Increasing the FOV often means accepting less resolution and a diminished image quality. Therefore, a more practical approach is to develop an optics system that can scan along the fibre during or between experimental phases and that can be actuated to compensate for stretch‐induced displacements of the FOV.

To address these limitations, we developed an upgraded biomechatronics platform: the *MyoRobot 3.0*. This next‐generation device integrates a high‐performance microscopy system mounted onto a two‐axis motorized stage, allowing targeted scanning across the fibre–s length, while being mechanically uncoupled from the biomechatronics stage. This implementation overcomes previous structural imaging constraints, capturing comprehensive structural information before, between, or after mechanical recordings. These scans can be stitched together to reconstruct full‐length views of individual muscle fibres at sarcomere resolution.

In this manuscript, we describe the technical development and implementation of this opto–mechanical integration and demonstrate its added value through proof‐of‐concept experiments. In these, we show how fibre diameter narrows considerably during stretch – on average by ∼13%–15% for 40% strain – a phenomenon that is often neglected in literature [13, 25, 26]. Assuming a constant fibre diameter during stretch results in a systematic underestimation of restoration stress by roughly 25%–28%, which we corroborate both experimentally and analytically using a cylinder model.

Furthermore, with this improved structural analysis capability, we revisited and compared two established experimental protocols for assessing passive (visco‐)elastic fibre properties: stress–strain recordings [26, 27, 28] and stepwise “stretch‐jumps” or “stress‐relaxation” [13, 17, 25, 28, 29]. We observed that even slow, continuous stretches at 1 μm/s result in a still marked viscous contribution to restoration stress, suggesting that stress–strain relationships may not be ideally suited to delineate elastic components. In contrast, stepwise “stretch‐jumps” – which allow prolonged adaptation periods at discrete stretch intervals – appear more appropriate for quantifying the purely elastic response.

Together, these findings establish the *MyoRobot 3.0* as a powerful tool for integrated optical and mechanical muscle fibre analysis and underscore the importance of resolving structural heterogeneity to enhance the accuracy and reproducibility of single muscle fibre biomechanics.

## 2 Materials and Methods

### 2.1 Animals and Muscle Fibre Preparation

All procedures were conducted in accordance with animal welfare regulations approved by the Friedrich–Alexander–University Erlangen–Nürnberg (TS06/2016), following German and European animal research guidelines. Tissue samples were obtained through a local tissue‐sharing collaboration and consisted of male C57BL/6 mice (4‐6 months, ∼30 g body weight).

The *M. Extensor Digitorum Longus* (EDL) muscle was rapidly excised from the hindlimb of a mouse under a stereomicroscope. Muscle isolation was done in Ringer–s solution containing (in mM): 145 NaCl, 5 KCl, 2.5 ${\mathrm{CaCl}}_{2}$, 1 ${\mathrm{MgCl}}_{2}$, 10 glucose, and 10 HEPES ((4‐(2‐hydroxyethyl)‐1‐piperazineethanesulfonic acid), pH adjusted to 7.4 using 1 M NaOH). To permanently depolarise the membrane and arrest the muscle in a relaxed state, Ringer's solution was exchanged for high‐potassium solution containing (in mM): 140 K‐glutamate, 1 ${\mathrm{MgCl}}_{2}$, 10 HEPES, 1 EGTA (ethylene glycol‐bis(β‐aminoethyl ether)‐N,N,N',N'‐tetraacetic acid), 10 glucose (pH adjusted to 7.0 using 1 M NaOH). The sample was kept on a cooling plate between 6
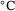
–8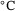 for 30 min prior to single fibre isolation, allowing a thorough diffusion of the sample with high‐potassium solution.

Single fibres were manually dissected and tied to micro‐knots at each end using fine braided silk thread (9‐0 suture, Pearsalls Ltd). The fibre was then mounted to the *MyoRobot* between a voice coil actuator (VC, CAL12‐010‐51‐1, SMAC Corporation) and a force transducer (FT, KG7B, 0‐10 mN, 1 μN resolution, World Precision Instruments). During fibre mounting, the specimen was kept in high‐potassium solution. Once attached, a vertical motor lifted the fibre out of the solution and lowered it into a well with relaxing “idle” solution (see below).

### 2.2 Bioactive Solutions Used in *MyoRobot* Experiments

All intracellular‐like solutions were adjusted to pH 7.0 at 22
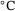
–23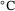 (room temperature), and all experiments were conducted under the same ambient conditions. Force and diameter data from each fibre were acquired at 10 Hz, except for the stepwise “stretch‐jump” protocol acquiring data at 100 Hz.

#### 2.2.1 High Activating Solution (HA)

High activating solution provides a calcium‐saturated environment with a calculated free [${\mathrm{Ca}}^{2+}$] of 12 μM (pCa 4.92, Table 1). It was used to probe maximum contractile force prior to passive biomechanics assessment and to investigate calcium‐dependent modulations of passive stiffness.

**TABLE 1:** Solution compositions used for fibre experiments. All concentrations are given in mM.

Substance	HA	HR	LR
HEPES	30	30	30
Mg(OH) 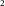	6.05	6.25	7.86
EGTA	30	30	0.4
${\mathrm{CaCO}}_{3}$	29	—	—
HDTA	—	—	6.6
K‐Glutamate	—	—	87.7
${\mathrm{Na}}_{2}\mathrm{ATP}$	8	8	8
${\mathrm{Na}}_{2}\mathrm{CP}$	10	10	10

#### 2.2.2 High Relaxing Solution (HR)

High relaxing solution creates a calcium‐free environment with a pCa of ∼9.0 (Table 1). The inclusion of 30 mM EGTA ensures robust calcium chelation, thereby providing a highly buffered environment that effectively binds residual ${\mathrm{Ca}}^{2+}$ ions in the internal solution. HR was used to fully relax skinned fibres and served as the baseline condition for passive mechanics. It was also reintroduced after exposure to ${\mathrm{Ca}}^{2+}$‐containing solutions to re‐establish ${\mathrm{Ca}}^{2+}$‐free conditions prior to subsequent experimental steps.

#### 2.2.3 Low Relaxing Solution (LR)

This solution also provides a nominally calcium‐free environment, using the low‐affinity calcium buffer HDTA (6.6 mM) in combination with a minimal amount of EGTA (0.4 mM, Table 1). It is introduced whenever the fibre was previously immersed in a high‐EGTA solution, such as HR, to exchange the high‐affinity buffer for a low‐affinity one. This is essential to prevent excessive chelation of calcium ions during subsequent calcium exposure.

Both, HA and HR solutions contain strong calcium buffers and are initially acidic due to their phosphate‐free but chelator‐rich composition. To achieve the target pH of 7.0, both buffers required titration with ∼90 mM KOH. This base addition is essential to bring the solution into physiological pH range. Consequently, the adjusted ionic strength of HA and HR markedly differs from that of LR solution. LR contains ∼87.7 mM K‐glutamate, a weakly basic anion that compensates for the KOH‐derived ionic load in HA/HR. Glutamate, being a zwitterionic amino acid near physiological pH, contributes to the buffering capacity by donating or accepting protons in response to pH changes [30].

#### 2.2.4 Idle Solution

For SL calibration and initial fibre diameter recordings, a diluted intermediate ${\mathrm{Ca}}^{2+}$‐free solution (termed “idle”) was used. This was prepared by mixing 1000 μL LR solution with 5 μL HR solution.

#### 2.2.5 Intermediate Calcium Solution (pCa 6.03)

To investigate passive fibre mechanics under moderate calcium levels, an intermediate solution with a calculated pCa of 6.03 (∼1 μM) was prepared by mixing high activating and high relaxing solutions in a 7:3 volume ratio. This setup permits exploration of half‐maximal calcium‐mediated effects on fibre stiffness.

#### 2.2.6 BTS‐Containing Solutions

To inhibit acto‐myosin cross‐bridge cycling and to isolate passive properties, N‐benzyl‐p‐toluene sulfonamide (BTS) was added to the respective solutions at a final concentration of 20 μM. BTS was included in selected experiments across three calcium conditions:

- Relaxing (pCa 9.0 ‐ 100% HR)
- Intermediate (pCa 6.03 ‐ 70:30 of HA to HR)
- Activating (pCa 4.92 ‐ 100% HA)

### 2.3 MyoRobot 3.0 Opto–Biomechatronics Platform

**FIGURE 2:**
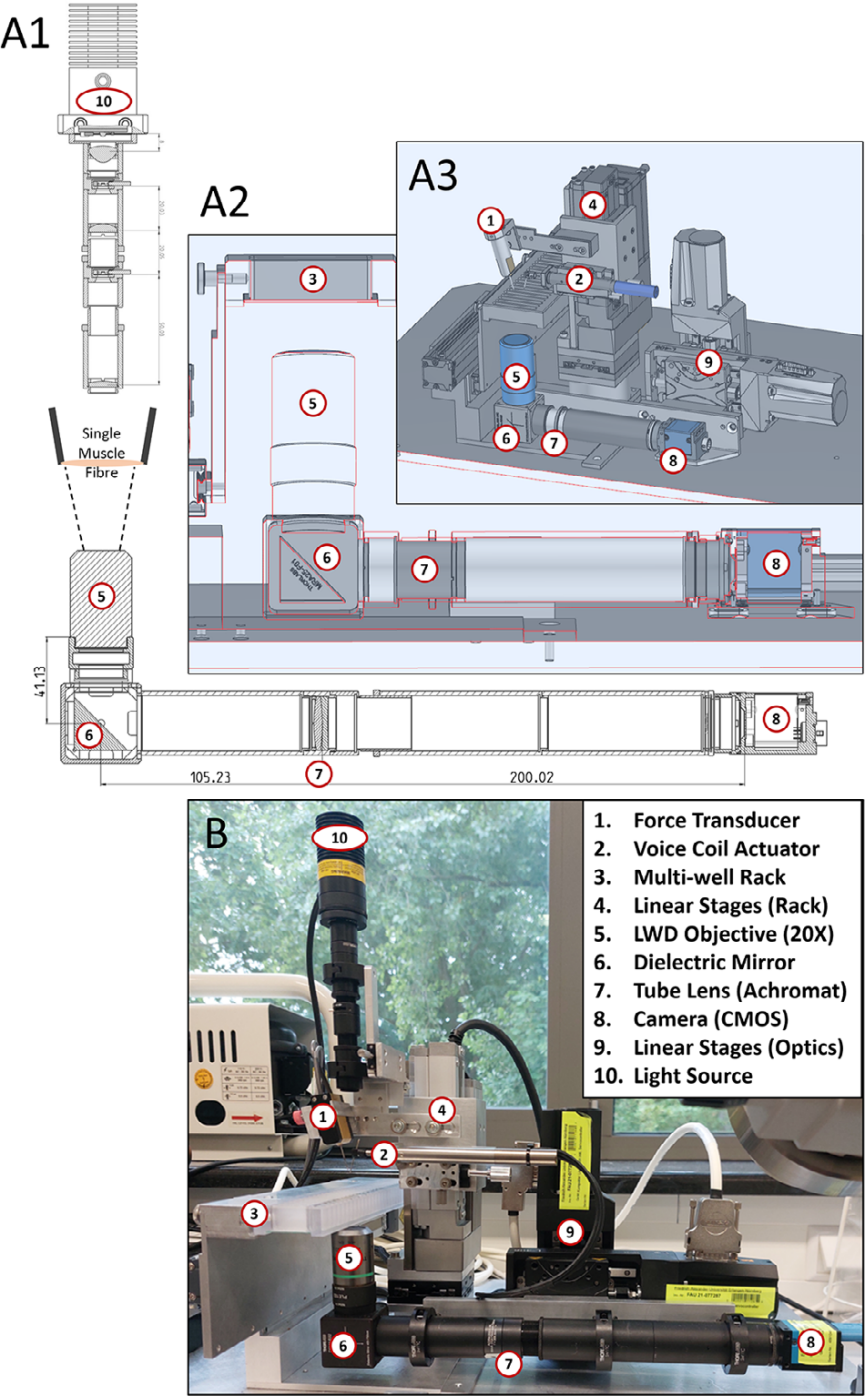
Overview of the Integrated Optics System. (A1) Technical drawing showing a cross‐section of the entire beam path. A light source (10) provides Köhler illumination to the sample plane which is picked up by the objective (5) below, deflected by a mirror (6) and focused onto the camera (8) by a tube lens (7). Measures given in mm. (A2) Displays the imaging system below the rack (3) in which the muscle fibre will be immersed. (A3) Shows the entire CAD drawing of the *MyoRobot 3.0*, including the VC (2) and FT (1) between which the sample is mounted. The entire imaging unit is attached to two cross‐stages for motion control. (B) Photograph of the *MyoRobot 3.0* including all key‐components.

The *MyoRobot* (as previously described in Ref. [31]) is an opto–biomechatronics platform engineered for comprehensive structural and functional analysis of single muscle fibres (Figure 2). While upstream‐compatible with fibre bundles and whole muscle preparations, its architecture is optimized for single‐fibre applications. It features a 35‐well rack which allows automated exposure of the mounted fibre to various bioactive solutions for inducing contractile or relaxation states. The system employs a high‐resolution piezoelectric FT to record active and passive forces, paired with a high‐precision VC to apply defined stretches. The previous iteration (*MyoRobot 2.0*) included a manually adjusted transmission optics with an open beam path, illuminated by a gooseneck lamp. This setup was much prone to scattered light, which compromised image quality and ultimately, signal‐to‐noise ratio. These limitations, together with the FOV constraints mentioned above, prompted us to conceive the development of an enhanced optics system within the new *MyoRobot 3.0*.

Central to this mechano–optical engineering endeavour is a dual‐axis motorized linear stage that supports automated scanning of the muscle fibre during resting phases or between protocol steps. The linear drives (L‐406, Physik Instrumente GmbH) enable precise step sizes as small as 0.5 μm, with a travel range of up to 26 mm, adequate to cover the full length of murine EDL single fibres. A Köhler illumination system was engineered to produce homogenous, high‐contrast illumination across the specimen plane. The muscle fibre is imaged through a long working‐distance, infinity‐corrected objective (Nikon TU Plan Epi ELWD 20×, NA 0.4, WD 19 mm), a deflection mirror, a 200 mm focal length tube lens with anti‐reflective coating (AC254‐200‐A‐ML, Thorlabs), and a CMOS camera sensor (DFK 33UX250, The Imaging Source) with 2,448×2,048 pixels at 3.45×3.45 μm pixel size. This provided a FOV covering

∼360 μm of a single fibre segment, which typically included ∼130–150 individual sarcomeres. All optics components are enclosed in a sealed tubing assembly to eliminate stray light. The light source (MWWHL4, Thorlabs) exhibits two spectral peaks at 450 and 600 nm, resulting in theoretical resolutions of

$r_{450\;nm}∼$562.5 nm and $r_{450\;nm}∼$750.0 nm, respectively. Therefore, the setup provides a 20× magnification and resolution sufficient to resolve sarcomere structures known to be at least 1 μm in size [32, 33]. The imaging system is controlled through LabVIEW‐based software that coordinates axial scanning, focus, and operation during stretch experiments.

### 2.4 Second Harmonic Generation (SHG) Imaging

To cross‐validate sarcomere length estimations, fixed single fibres previously measured in the *MyoRobot* 3.0 were subsequently imaged using *Second Harmonic Generation* (SHG) microscopy. After completing the mechanical recordings, the same fibres were fixed in 4% paraformaldehyde (PFA) to preserve their structural integrity and were then prepared for SHG imaging. SHG is a label‐free imaging technique that leverages non‐linear optical properties of non‐centrosymmetric structures–such as myosin and collagen–to generate contrast without involving fluorescent dyes [2, 34, 35]. Given that the isolated single muscle fibres were largely devoid of extracellular matrix, the dominant SHG signal originated from the periodic myosin arrangement within sarcomeres.

For excitation, a pulsed infrared laser at 810 nm was used, and the emitted SHG signal was isolated using a 405 nm bandpass filter. Raster scanning across the fibre generated 3D image stacks with a lateral resolution of 1024×1024 pixels and a physical area of 409×409 μm per slice. Z‐stacks were collected in 1 μm steps along the radial axis of the fibre. The forward‐propagating SHG signal (transmission) was detected using a photomultiplier tube, as this component predominantly represents myosin‐specific signal, whereas back‐scattered reflection tends to originate from collagen structures [36, 37].

Image analysis was performed using a custom Fiji‐based processing pipeline [38]. Each optical slice was processed via 2D Fourier transformation, filtered, and analyzed for periodicity to calculate sarcomere length. Results were averaged across the Z‐stack to yield a representative mean sarcomere length per fibre, along with standard deviation. These values were statistically compared against those obtained by the *MyoRobot* software using Shapiro‐Wilk tests for normality, followed by Kruskal–Wallis ANOVA and Tukey post‐hoc tests for multiple comparisons.

### 2.5 MyoRobot 3.0 ‐ Passive Biomechanics Recording Protocols

Before conducting any functional measurements, muscle fibres were adjusted to an average SL of 2.5 μm (close to the descending limb of the optimal actin‐myosin overlap [39]). This adjustment was performed within the current FOV, after which the initial fibre diameter was calculated using established algorithms [31] to compute the circular cross‐sectional area. This localized approach (obtaining the fibre diameter on a distinct axial location) was used in cases where functional characterization was prioritized, since automated routines for scanning the entire fibre are still under development.

In contrast, when structural parameters (such as sarcomere length or diameter distribution along the full fibre axis under various stretch conditions) were the focus, the objective lens was moved along the entire fibre length, with re‐focusing performed as needed to capture a continuous image dataset of the fibre diameter distribution.

To allow diffusional access to the cytosol and enable skinned fibre experiments, the sarcolemma was permeabilized by treating the fibres with 0.01% (w/v) saponin in high relaxing solution for 20 s.

Each experiment probing for different conditions (e.g., comparison between stretch protocols, or presence/absence of BTS) was performed in a sequence on an individual fibre preparation to allow for a pairwise comparison.

#### 2.5.1 Resting Length‐Tension Curves (RLT) / Stress–Strain Recordings

To assess the elastic biomechanics properties in axial fibre direction, single muscle fibres were stretched by 40% of their initial length ($L_{0}$) at a constant speed of 1 μm/s, while force was continuously recorded at 10 Hz. The amount of time required to perform this stretch was computed automatically. To ensure the same muscle fibre segment remained in the FOV during stretch protocols, the velocity of the motorized optics system was computed. Since the fibre is stretched between a static FT and a moving VC pin, the stretch is not uniform across the fibre–s length. The relative displacement of a segment depends on its position along this axis and computes to:

(1) $$v_{optics}=\frac{|FT-OP|+180.15\;\mu m}{\left|VC-FT\right|}*v_{stretch}$$

Where:

- FT = coordinate of the FT pin
- VC = coordinate of the VC pin
- OP = starting coordinate of the optics system
- $v_{stretch}$ = stretch velocity applied by the VC
- 180.15 μm = correction term (half the FOV)

This allowed to continuously track the diameter change of the very same fibre segment during the RLT recording. To convert forces to stress, the cross‐sectional area (CSA) was computed based on the diameter data assuming a circular CSA. Stress–strain relationships were constructed from time‐resolved force and diameter recordings obtained during stretch protocols. Fibre diameter was continuously tracked via edge detection at 10 Hz and subsequently smoothed as described above. At each time point, the cross‐sectional area (CSA) was calculated assuming a circular geometry, and stress was computed as the ratio of force to CSA. This approach enables dynamic correction of stress values during stretch by accounting for diameter changes at the corresponding strain level (e.g., force at 27% strain normalized to CSA at 27% strain). The Young's modulus was eventually computed from the linear slope of the stress–strain curve, whereas the maximum restoration stress describes the maximum stress at 40% strain.

#### 2.5.2 Stepwise “Stretch‐Jumps” / “Stress‐Relaxation” Curves

Stepwise stretch‐jump protocols involved instantaneous length increases of 10% $L_{0}$ (at 250 mm/s) up to 40% $L_{0}$ in four steps (force data sample rate: 100 Hz). Between stretches, fibres were held at the new length for 60 s to allow for viscous stress relaxation. The steady state stress was obtained from the plateau after viscous relaxation, as it describes the elastic contribution [13]. The 60 s relaxation period was chosen as a compromise between allowing sufficient viscoelastic stress relaxation and maintaining feasible experimental durations. While shorter hold phases (5–10 s) are commonly used [13, 17, 40], they may not fully capture slower relaxation processes. Conversely, longer relaxation times, e.g. 50‐60 s [41] may further approach steady‐state conditions. Therefore, 60 s was considered a practical and biologically meaningful approximation of the elastic plateau. Plotting the elastic stress vs. the respective strain allows to compute the Young's modulus by applying a linear fit. This enables a direct comparison with the stress–strain‐derived values from nominally quasi‐static length‐tension curves.

All representative stress–strain traces correspond to individual fibres unless otherwise stated. Group data were derived from independent fibres and are reported as mean ± SEM.

### 2.6 Fibre Diameter Smoothing Algorithm

Fibre diameter was tracked during functional recordings using an edge‐detection routine operating at approximately 10 Hz. This algorithm continuously analyzed the live video feed to detect the fibre boundaries within the current field of view and stored the resulting diameter values as numerical time‐series array. Aside from a single recorded reference image–used to document average sarcomere length and diameter at the beginning of each experiment–all post processing (e.g., stress computation) was performed on the numerical time‐series array.

**FIGURE A1:**
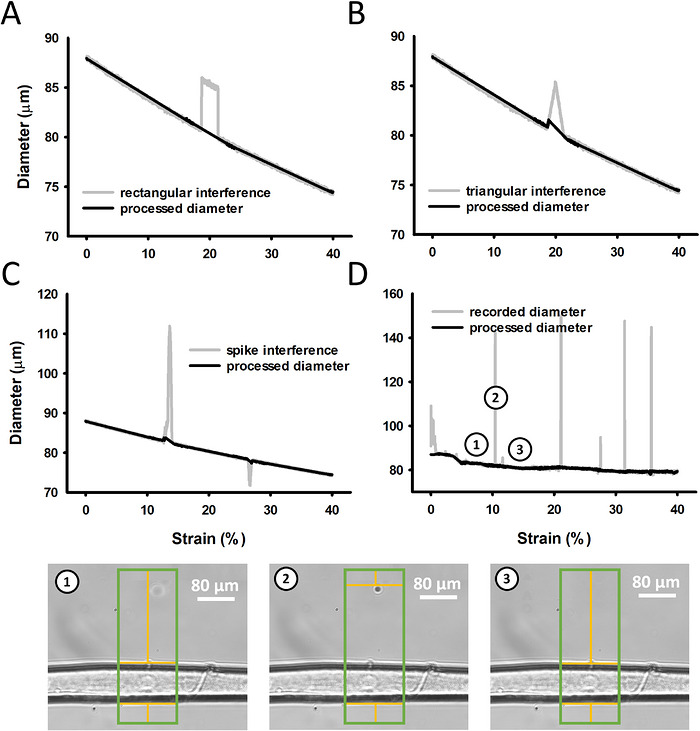
Handling artifacts in real‐time diameter tracking using a dedicated smoothing algorithm. **(A–C**) Simulated fibre diameter traces (grey) were overlaid with artificial artifacts of different profiles: **(A**) rectangular; **(B**) triangular; **(C**) spike‐shaped. In all cases, the processed traces (black) show accurate reconstruction of the underlying trend. **(D**) Application of the same algorithm to an actual muscle fibre recording. The raw data (grey) exhibit multiple high‐amplitude artifacts, likely due to debris or sedimentation in the open *MyoRobot 3.0* well. After filtering (black), the trace reflects the physiologically expected gradual decrease in diameter during stretch. Insets 1‐3 exemplarily display the occurrence of 'spikes' in the diameter trace. At some stage (1), a particle may sediment into the detection area (green rectangle). For the short time in focus (2), this particle is interpreted as obstacle, instantaneously but transiently increasing the computed diameter. Once past the focal plane (3) the actual fibre diameter continues to be recorded.

During long‐term biomechanics experiments, it is, however, unavoidable that small dust particles, remnants of the silk thread, or residual tissue debris occasionally enter the field of view. These artifacts may transiently interfere with edge detection (see Figure A1), resulting in local discontinuities or skewed diameter readings ‐ reflected in sharp data spikes, plateaus or other artifacts in the time‐series array. To minimize their impact on stress calculations, all raw diameter traces were processed during data analysis using the following algorithm:

1. The first few diameter values of the time‐series array are set to the initial diameter obtained from the reference image. This suppresses diameter fluctuations recorded during the initial solution/well exchange. Diameter values from the array are continued to be used once the measured values match or fall below the initial reference diameter.
2. A moving average and moving standard deviation are calculated using both left‐directed (past values) and right‐directed (future values) windows. The window size and a user‐defined z‐score threshold can be specified in the software. For each data point, the algorithm computes:
  An upper threshold: U=m+zs
  A lower threshold: L=m−zs
*Where: m = moving average; s = moving standard deviation; z: user‐defined z‐score (default: 1.5)*
  Both thresholds are computed twice–once using left‐directed and once with right‐directed windows. The algorithm selects the stricter of both boundaries. Values outside these boundaries are classified as outliers and temporarily replaced with “NaN”.
3. All NaN (outlier) values are linearly interpolated from the neighbouring diameter values to reconstruct a clean, gap‐free diameter array that matches the original time series length.
4. The interface displays both, the raw and processed diameter traces for user evaluation. If needed, users can adjust the z‐score or window size to fine‐tune the filtering.
5. The processed diameter trace is used to calculate stress (force/area). Stress–strain diagrams are saved as image files (BMP), and all processed data are exported as TDMS format. For further post‐processing please refer to the Appendix (p.15).

**FIGURE 3:**
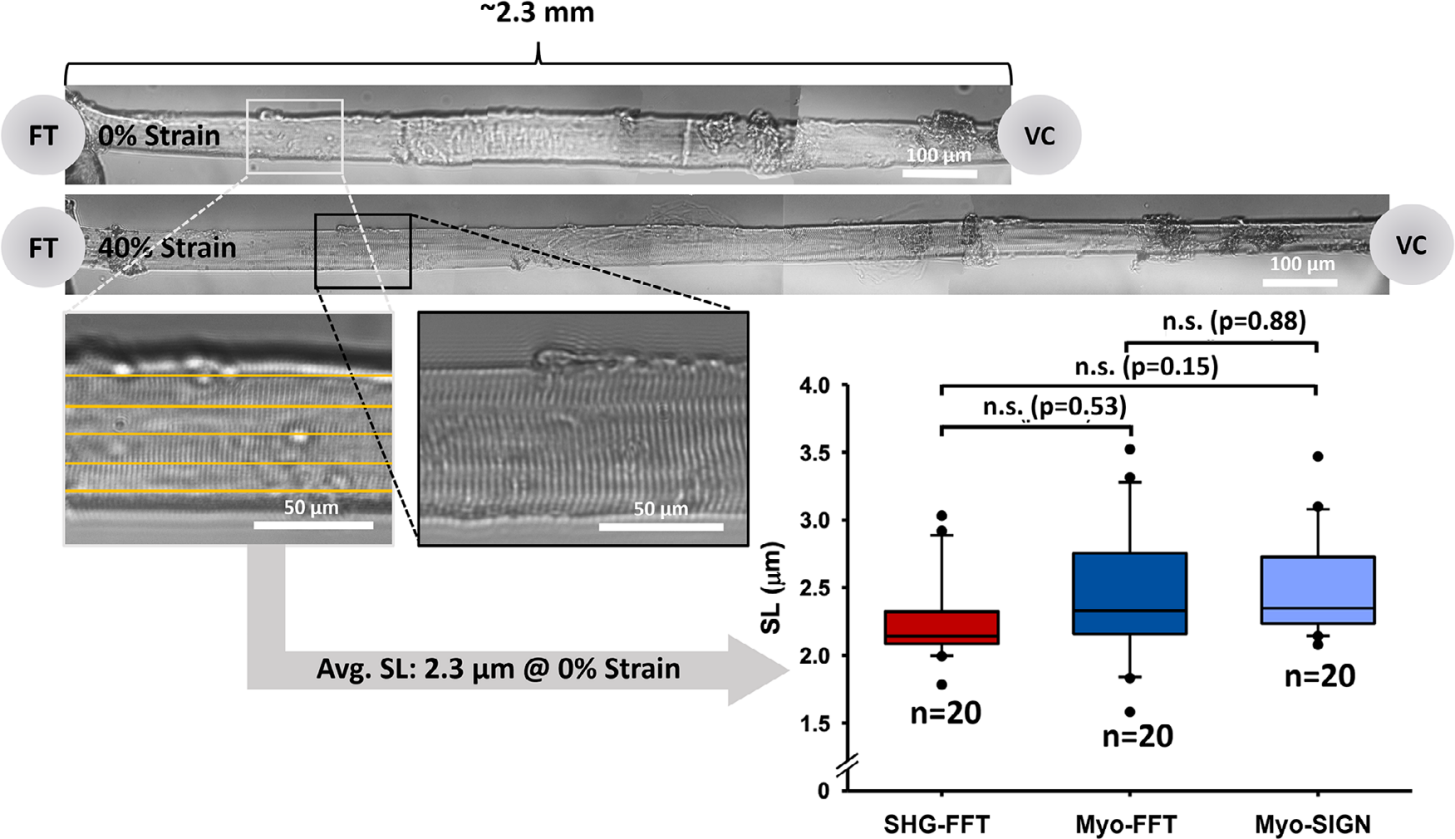
Quantitative comparison of average SL values obtained from stitched brightfield images. Top: Brightfield image stitching of a single EDL muscle fibre at 0% ($L_{0}$) and 40% strain. Insets highlight preserved sarcomere detail under stretch. **Bottom right**: Comparison of SL values obtained using three analytical pipelines: SHG‐based FFT algorithm (SHG‐FFT), Myo‐FFT, and Myo‐SIGN (algorithms described in Ref. [31]). Data from n = 20 fibres show large agreement across methods, confirming the accuracy of the *MyoRobot 3.0* imaging and analysis pipeline.

**FIGURE 4:**
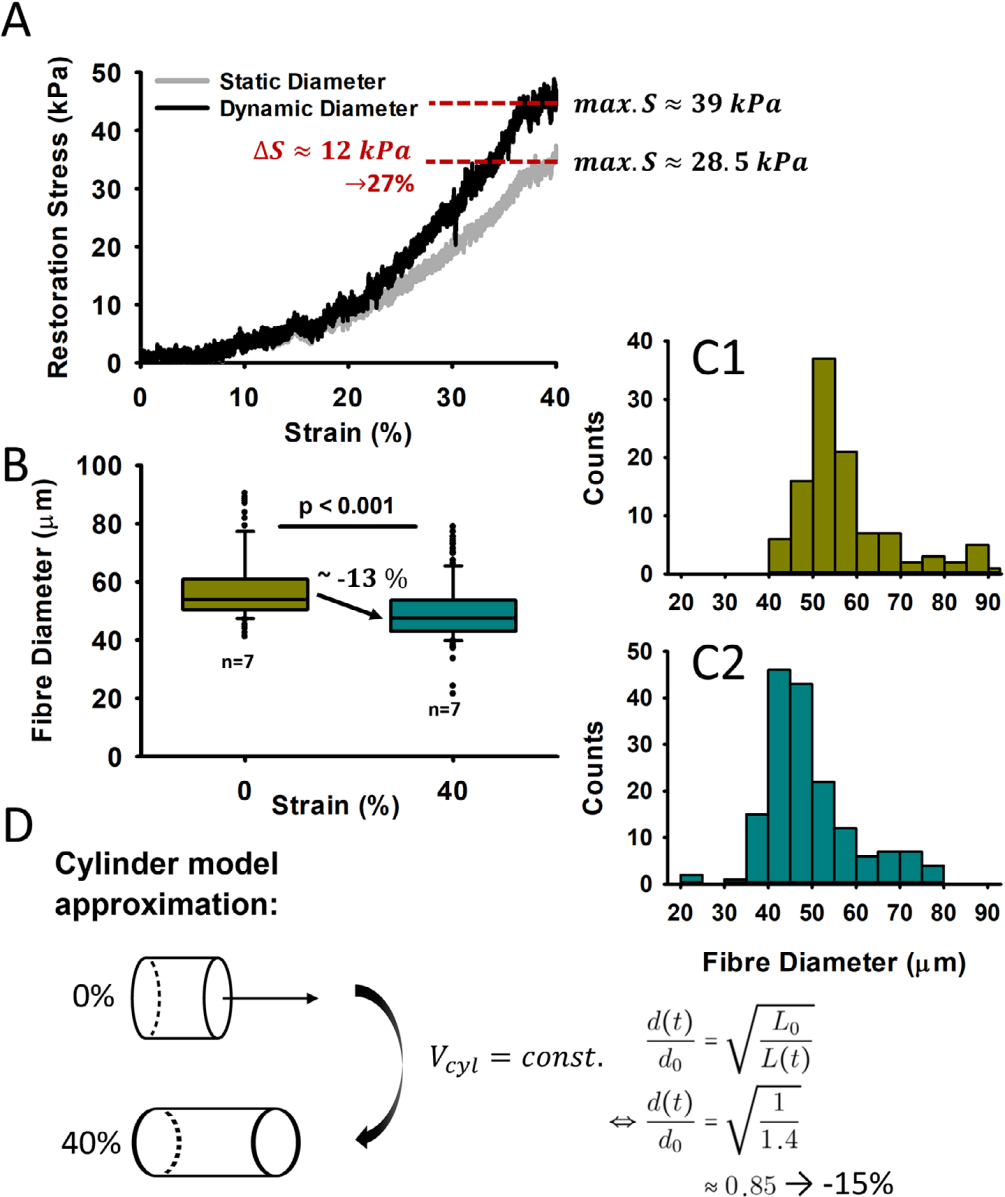
Fibre thinning during stretch causes stress underestimation unless dynamically accounted for. (A) Representative stress–strain recording comparing restoration stress based on either a static (gray) or dynamic (black) fibre diameter. At 40% strain, ignoring diameter thinning leads to ∼27% underestimation (Δ S∼8 kPa). (B) Boxplots showing the average fibre diameter of seven fibres at 0% and 40% strain, highlighting a significant reduction of ∼13%. (C1,C2) Corresponding diameter histograms across the full fibre length confirm diameter thinning under stretch. (D) Approximation using a cylinder model predicts a 15% diameter reduction at 40% strain, leading to a 15% diameter and thus, 27.75% area loss–mirroring the empirically observed stress deviation in (A).

**FIGURE 5:**
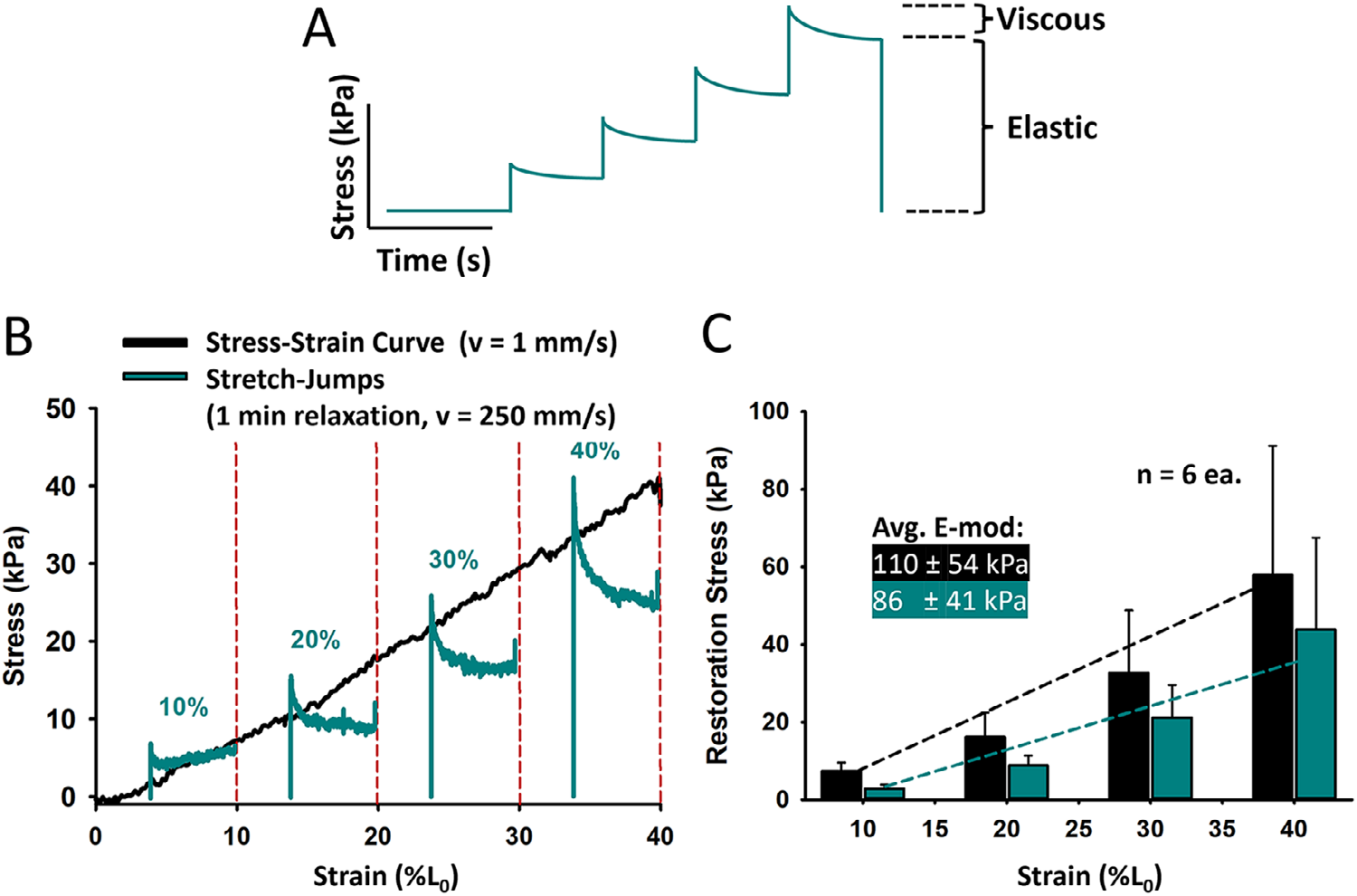
Comparison of stress–strain vs. stepwise‐stretch recordings for assessing axial elasticity in single muscle fibres. (A) Schematic of the stretch‐jump protocol. The fibre was stretched in four discrete steps of 10% $L_{0}$, each followed by a ∼1‐min relaxation phase to allow for visco–elastic stress decay. (B) Representative comparison between a continuous stress–strain recording (black) and a stepwise stretch‐jump protocol (teal). The stress–strain curve is plotted as a function of continuously increasing strain. In contrast, the original stretch‐jump recording is time‐based and consists of discrete stretch events followed by relaxation phases. For visual comparison, the stepwise recording was segmented and aligned to corresponding strain levels (10%–40%), such that the steady‐state plateau stress after each relaxation phase is mapped to the respective strain (highlighted by teal labels). The apparent discontinuities and non‐continuous progression of the teal trace arise from this transformation of time‐based data into strain space and do not reflect interruptions in the original recording. Red dashed lines indicate the strain levels at which plateau stresses from the stretch‐jump protocol were compared to the corresponding values in the continuous stress–strain curve. (C) Quantification of elastic restoration stress from six fibres with both protocols implemented for each fibre. Mean values and standard errors are shown. The derived Young's modulus from linear fits was markedly lower for stepwise‐stretch recordings (not significant), suggesting the continuous 1 μm/s to still contain viscous contributions during stretch.

**FIGURE 6:**
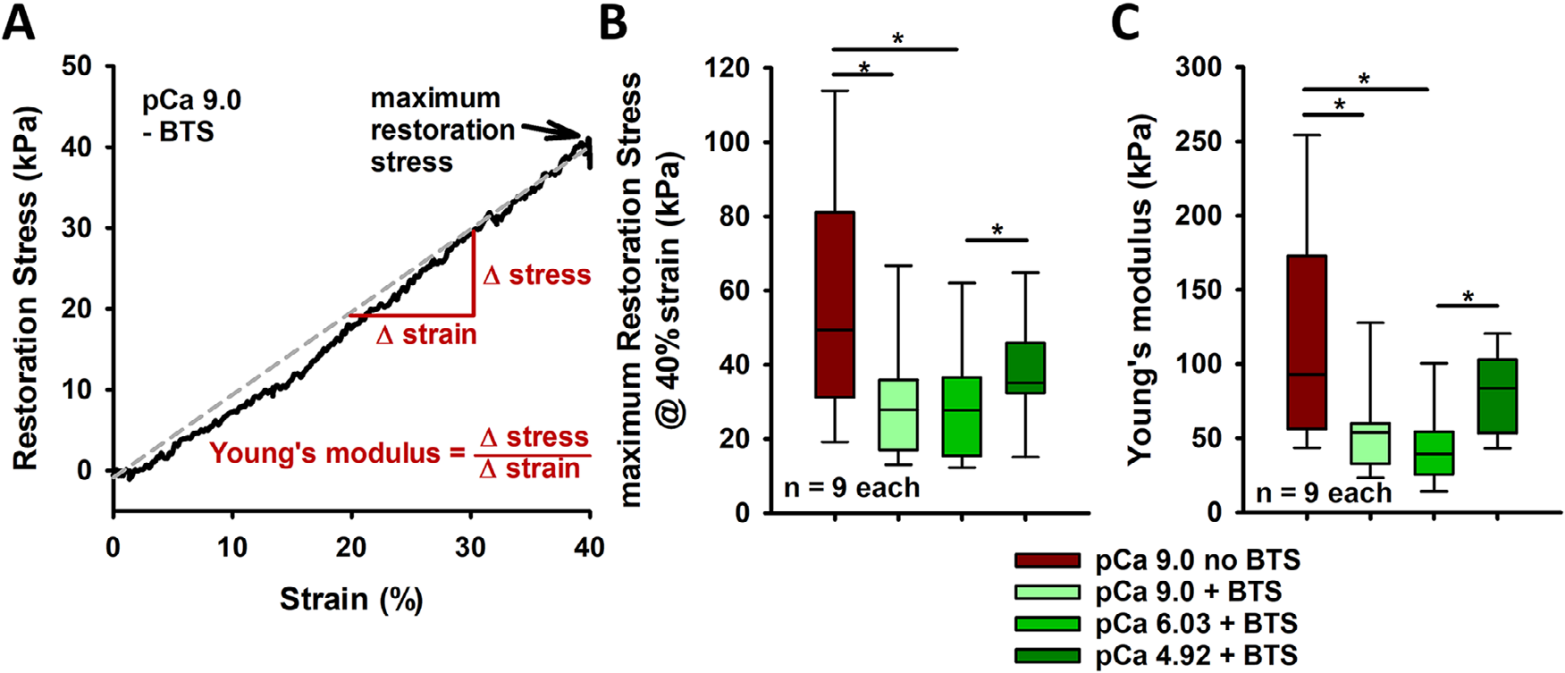
Calcium‐dependent modulation of passive restoration stress and Young's modulus in chemically skinned fibres. (A) Representative stress–strain trace used to derive restoration stress and Young's modulus. (B) Maximum restoration stress at 40% strain across the four conditions. (C) Young's modulus comparison across the same four conditions. Data were obtained from the same fibres across all conditions. Asterisks indicate statistical significance (p <0.05). Note that the highest stiffness was observed in control fibres (no BTS), and significant calcium‐dependent stiffening was seen under cross‐bridge block at pCa 4.92.

**FIGURE 7:**
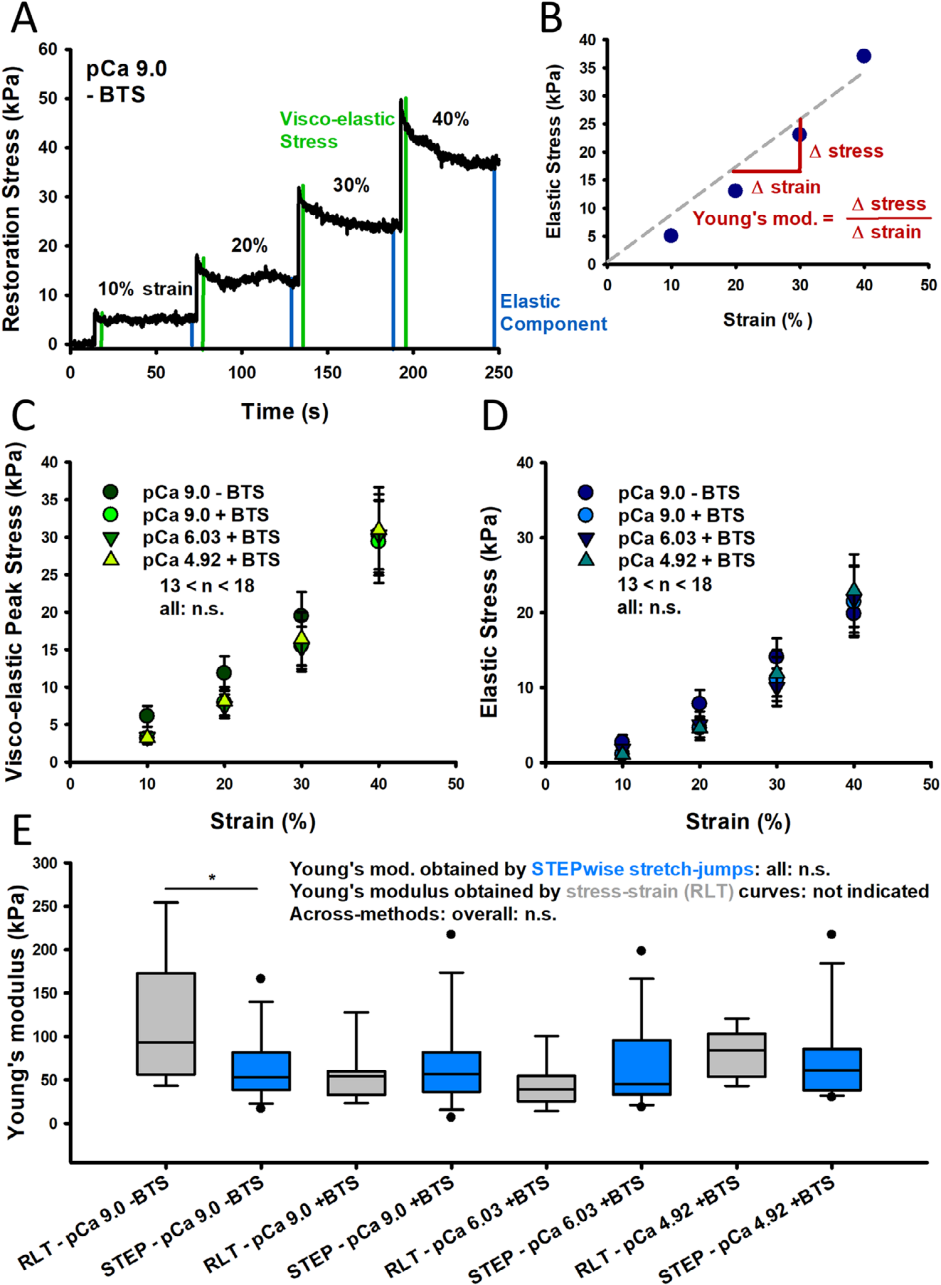
Calcium‐dependent changes in passive stress characteristics differ depending on stretch protocol. (A,) Representative trace of a single fibre subjected to stepwise “stretch‐jumps” under calcium‐free conditions (pCa 9.0, ‐BTS). Vertical lines mark the visco–elastic peak stress (green) and the elastic plateau stress (blue), the latter measured after stress relaxation. (B) Exemplary calculation of Young's modulus from a linear fit to elastic stress values plotted against strain. (C) Grouped data of visco–elastic peak stress; all p >0.05 within each strain level. (D) Elastic stress values after stress relaxation (1 min); all p >0.05 within each strain level. (E) Comparison of Young's moduli obtained from stress–strain curves (grey) and stepwise “stretch‐jumps” (blue). No group‐level differences were significant across conditions. A significantly higher Young's modulus was detected in the control group (pCa 9.0 ‐BTS) when derived from stress–strain recordings.

### 2.7 Investigating Passive Biomechanics under varying Calcium Conditions

To examine the calcium dependence of passive elasticity, fibres were subjected to RLT/stress–strain and stepwise stretch‐jump protocols in the presence or absence of 20 μM BTS at three defined calcium concentrations resulting in the following study conditions:

- pCa 9.0 (no BTS): standard relaxing condition
- pCa 9.0 (+BTS): relaxed condition with BTS, blocking residual acto‐myosin interaction [42]
- pCa 6.03 (+BTS) intermediate calcium levels with active contraction blocked
- and pCa 4.92 (+BTS): ‘saturated’ calcium levels with active contraction blocked

### 2.8 Statistical Analysis

Statistical analyzes were performed using Sigma Plot (previously: Systat ‐ now: Grafiti). Data were first tested for normality using the Shapiro–Wilk test. Depending on the outcome, either parametric or non‐parametric tests were applied.

For comparisons across multiple conditions, repeated measurements were obtained from the same individual muscle fibres. Therefore, where applicable, paired statistical approaches were used. For normally distributed data, repeated‐measures one‐way ANOVA was performed, followed by Holm–Sidak or Bonferroni *post‐hoc* tests. For non‐normally distributed data, the Friedman test was used with appropriate *post‐hoc* multiple comparisons.

Data are presented as mean and SEM. The number of biological replicates (individual fibres) is indicated in the respective figure legends. Statistical significance was accepted at p<0.05.

## 3 Results

### 3.1 Validation of Structural Imaging Capabilities

A major limitation of earlier biomechatronics setups, including the previous *MyoRobot 2.0* system, was the inability to acquire structural information along the full length of long single muscle fibre segments (∼2 mm). Due to a fixed optical FOV and static image acquisition, only a small, localized segment (∼150μm) could previously be imaged, introducing a risk of sampling bias and misinterpretation.

To overcome this bottleneck, the *MyoRobot 3.0* has been equipped with a newly engineered cross‐motorized optical system, enabling scanning and image stitching along the full fibre segment length. Figure 3 illustrates this capability, displaying stitched brightfield images of a representative fibre at resting length (0% strain) and under stretch (40% strain). Insets highlight the preserved image clarity and structural detail, underscoring the robustness of the optical and mechanical integration. Using the individual images of the overall stitched image, SL was quantified in five longitudinal sections distributed along the length of the muscle fibre in each individual image (as indicated by the yellow lines in the inset). For this, the derivative of the intensity profile along each line was processed using either a signum function (“Myo‐SIGN”) or using fast Fourier transform (“Myo‐FFT”, for details see Ref. [31]).

To validate the accuracy of these approaches, SL values obtained from the stitched images were benchmarked against 3D SHG z‐stacks ‐ processed via a well‐established 2D‐FFT sarcomere length detection algorithm (“SHG‐FFT”, [38]). Boxplot comparisons from 20 individual fibres (Figure 3, lower right) show that both, Myo‐FFT and Myo‐SIGN deliver highly comparable results to SHG‐FFT, with no statistical difference observed. This validation confirms the system's capability to provide reliable, fibre‐wide structural information, enabling more accurate biomechanical parameter extraction and paving the way to explore more complex structure‐function relationship. It is noted that after structural fixation in 4% PFA in the *MyoRobot*, fibres were transferred to an SHG setup and imaged under the same passive length conditions.

### 3.2 Fibre Narrowing During Stretch Impacts Stress Estimation in Biomechanical Recordings

With the new cross‐motorized optics, we tackled a tacit assumption in single‐fibre biomechanics: a static / constant fibre diameter during stretch experiments. While convenient, this simplification introduces a systematic error in stress calculations, as stress is derived by normalizing force to CSA. With the improved optics system of the *MyoRobot 3.0*, we are now able to directly quantify diameter changes during, e.g., stress–strain recordings and assess how these affect restoration stress (Figure 4A).

To this end, full‐length scans of individual muscle fibres were acquired both, at $L_{0}$ (0% strain) and following 40% strain. Fibre diameters were extracted across the fibre's longitudinal axis (approx. each 50–100 μm, resulting in 20–40 diameter assessments in a 2 mm long single fibre) and are visualized as both, boxplots (Figure 4B) and histograms (Figure 4 C1,C2). This revealed an average diameter reduction of approximately 13% at 40% strain. Assuming a cylindrical geometry, another common assumption in muscle biomechanics, we obtained a theoretical reduction of 15% (Figure 4D) which aligns well with our empirical data.

To highlight the gravity of these findings on the computation of strain, we compared stress–strain curves obtained using either a static diameter assumption or dynamic diameter narrowing (example shown in Figure 4A). When accounting for diameter reduction during stretch, restoration stress was consistently higher, with differences reaching up to ∼12.5 kPa, or ∼27%, at 40% strain (Equation (2)). This value again aligns well with model‐based calculations and confirms that neglecting fibre thinning leads to a considerable underestimation of true restoration stress.

(2) $$\begin{array}{cc} \frac{A_{40\%}}{A_{0\%}} & =\frac{\pi *{\left(0.5*d_{40\%}\right)}^{2}}{\pi *{\left(0.5*d_{0\%}\right)}^{2}}=\frac{{\left(0.5*0.85*d_{0\%}\right)}^{2}}{{\left(0.5*d_{0\%}\right)}^{2}}\\ & =0.85^{2}=0.7225\to -27.75 \end{array}$$

### 3.3 Comparing Two Methods to Assess Axial Elasticity in Muscle Fibres When Considering Diameter Thinning

With reliable image‐based diameter detection, the *MyoRobot 3.0* system was applied to assess biomechanics properties of single skeletal muscle fibres under stretch. In particular, we evaluated how two common approaches for probing passive axial fibre stiffness – slow (nominally “quasi‐static”), continuous stress–strain recordings and fast (quasi‐instantaneous) stepwise stretch‐jump protocols Figure 5A – compare when fibre geometry dynamics is accounted for during stress computation.

Figure 5B shows a representative example for both protocols (or “stress‐relaxation” curves). The black trace depicts the continuous stress–strain curve, while the teal trace corresponds to the stretch‐jump protocol. Red dashed lines highlight the strain levels where the apparent elastic plateau stresses were extracted for comparison and computation of the Young's Modulus for every fibre. Intriguingly, both modalities yielded different passive restoration stresses and consequently, different Young's moduli (Figure 5C). At each stretch level, mean stress values were lower when extracted after visco–elastic adaptation in stepwise‐stretch recordings. This is also reflected in the average Young's modulus of ∼110 kPa (stress–strain) vs. ∼86 kPa (stepwise‐stretch), which is markedly larger when extracted from stress–strain relationships.

These results question the suitability of stress–strain recordings carried out at 1 μm/s to assess the purely elastic restoration stress and suggest that an even lower stretch velocity may need to be employed in such protocols. Considering an average fibre length of 2 mm, our stretch velocity translates to 0.05% $L_{0}$/s. Yet, stretching at even lower speeds (nm/s) will pose a marked technical challenge for continuous stretch protocols as the “long” (millimetres) actuation range of stretch controllers is opposed to a precise speed encoding (<1 μm/s). In the *MyoRobot*, 1 μm/s is the slowest true continuous actuation speed that can be governed by the encoder ‐ slower speed will result in a ratchet‐like movement. This favours the application of faster and more “high‐throughput” stepwise‐stretch jumps with prolonged visco–elastic adaptation periods.

### 3.4 Calcium Sensitivity of Passive Restoration Stress and Fibre Stiffness in Stress–Strain Recordings

With the *MyoRobot 3.0* being able to monitor diameter thinning during the experiment, we next explored how calcium modulates passive restoration stress, particularly under conditions where active force generation is blocked. Although our experimental setup does not allow to precisely discern the contributions of titin to a calcium‐dependent change in passive restoration forces, it is well‐known that titin contributes to and exhibits a calcium‐dependent stiffening [43, 44, 45]. Notably, this response may differ depending on the dynamics of stretch, with slow stretches (as applied here) mainly probing elastic behavior, while faster stretches also capture viscous contributions [46, 47, 48]. Therefore, we continued with comparing stress–strain recordings to stepwise “stretch‐jumps” to assess Young's modulus and restoration stress under four progressively calcium‐rich but cross‐bridge‐inhibited environments:

- Control (pCa 9.0, no BTS)
- pCa 9.0 + BTS (calcium‐free, S1 ATPase blocked)
- pCa 6.03 + BTS (moderate calcium, S1 ATPase blocked)
- pCa 4.92 + BTS (high calcium, S1 ATPase blocked)

A representative stress–strain trace is shown in Figure 6A, with the slope used to determine Young's modulus. Group analysis revealed that both, the maximum restoration stress at 40% strain (Figure 6B) and Young's modulus (Figure 6C) were significantly higher in control fibres (pCa 9.0 ‐ BTS) compared to the same fibres under myosin ATPase blockade (pCa 9.0 + BTS), underscoring the “softening” effect of BTS. Interestingly, elevating calcium concentration in the presence of BTS partially “restored” fibre stiffness. At pCa 4.92 + BTS, both, restoration stress and stiffness increased significantly relative to pCa 6.03 + BTS, pointing toward a calcium‐dependent stiffening of passive muscle fibres, even in the absence of acto–myosin cross‐bridge cycling. This supports the hypothesis that titin or other calcium‐sensitive elastic elements contribute to passive biomechanics in a calcium‐concentration‐dependent manner.

### 3.5 Passive Stepwise “Stretch‐Jumps” Reveal No Calcium‐Dependent Stiffening

After demonstrating that stress–strain recordings are sensitive to calcium concentration and the presence of myosin ATPase inhibition, we then applied stepwise “stretch‐jump” protocols to assess whether these results hold when the elastic restoration stress is computed after a prolonged period of viscous adaptation (Figure 7A). Each fibre was subjected to incremental stretch jumps, with each step followed by a 1‐min holding phase to allow for viscous relaxation. As highlighted in Figure 7A, this allowed to separate the peak stress and its relaxation kinetics immediately after the jump (mixed visco–elastic component) from the plateau stress after relaxation (pure elastic component). Based on this, the Young's modulus was calculated as the linear slope of strain vs. plateau elastic restoration stress (Figure 7B).

Interestingly, when comparing plateau restoration stresses across different ${\mathrm{Ca}}^{2+}$ and BTS conditions in this stepwise stretch protocol, no significant differences were observed for either the peak stresses (Figure 7C) or the plateau elastic stress (Figure 7D). These findings markedly contrast with the results derived from continuous stress–strain protocols, where condition‐specific differences were detectable. Therefore, we compared the Young's moduli derived from both recording strategies across all experimental groups

(Figure 7E). Again, the stepwise “stretch‐jumps” (blue) resulted in comparable stiffness across all test conditions ‐ contrasting the results obtained in stress–strain recordings (gray). However, in presence of BTS, we could not detect any statistically relevant differences between methods. The only significant difference across protocols was found for the control condition at pCa 9.0 without BTS, where the stress–strain protocol yielded a significantly higher Young's modulus compared to the stepwise approach. This supports the idea that continuous stretches at slow velocity (1 μm/s) may prolong weak acto‐myosin interactions, thereby leading to an increased stiffness when measured in the absence of BTS in ${\mathrm{Ca}}^{2+}$‐free conditions.

## 4 Discussion

This study introduces the *MyoRobot 3.0*, a next‐generation biomechatronics platform that integrates simultaneous structural imaging with mechanical testing to resolve a critical limitation in single‐fibre biomechanics: the inability to track structural changes across the full fibre length. Most systems (as the Aurora Skinned Fibre System [10] or the previous *MyoRobot 2.0* [31]) were restricted by a narrow, fixed optical FOV, which hinders accurate analysis of diameter changes, sarcomere variability, or fibre movement during stretch. In particular, in those mentioned systems, the optics remained stationary, while the sample had to be moved on the stage also containing force sensors and actuation pins. In our setup, the reverse condition was engineered with the optics being actuated during the stretch procedure. We address this gap by incorporating a cross‐motorized scanning optics system that enables fibre‐wide imaging before, between, or after biomechanics testing. This integration allows for dynamic measurement of structural parameters, more accurate stress calculations, and deeper insight into structure‐function relationships.

Still, a limitation of the present analysis lies in the assumption of a circular CSA for stress calculation. While this approximation is widely used in single‐fibre biomechanics [12, 13, 25], it does not account for potential anisotropy in fibre geometry. Skeletal muscle fibres deviate from a perfect circular shape and may exhibit elliptical or irregular cross‐sections depending on preparation geometry, mounting, and structural integrity [49, 50, 51]. In principle, more accurate CSA estimations could be achieved by assuming an elliptical geometry [52, 53]; however, this would require at least two orthogonal imaging planes (e.g., horizontal and vertical views [10]), which are not accessible (especially along the entire fibre length) in most standard biomechanics setups.

Importantly, even such multi‐axis approaches remain limited if CSA is only assessed at individual locations along the fibre. Our fibre‐wide scans revealed substantial longitudinal variability in diameter, indicating that CSA is not constant along the fibre axis. Consequently, single‐plane or single‐location measurements–regardless of whether circular or elliptical assumptions are applied–may lead to misrepresentation of the true CSA and thus of the resulting stress values. Recent work by Mebrahtu et al. [52], and Smith and Herzog [52, 53] has questioned the circular CSA approximation and proposed alternative geometrical interpretations. However, these approaches similarly rely on single‐plane imaging and do not account for longitudinal variability in CSA, which our data suggest is a major source of systematic error.

A comprehensive assessment of fibre geometry would, therefore, require tomographic [54] or multi‐angle imaging [10] combined with full‐length reconstruction, which is currently not compatible with real‐time biomechanical measurements and would likely require independent methodological developments. However, given the pronounced longitudinal variability in fibre diameter observed in our study, we consider dynamic diameter tracking to be the dominant factor for accurate stress estimation.

### 4.1 Stress is Underestimated When Fibre Thinning is Ignored

A central finding enabled by the *MyoRobot 3.0* is the quantification of fibre diameter thinning during axial elongation – a structural adaptation that directly affects mechanical outcome. Historically, single‐fibre biomechanics accepted the assumption that fibre diameter, and thus CSA, remains constant during stretch [13, 15, 55, 56]. With a motorized optics system capable of performing stretch corrections, we reveal a systematic diameter reduction of ∼13% at 40% strain, consistent with theoretical predictions from cylindrical volume conservation (∼15%). This structural change translates into a ∼27% reduction in CSA, which results in a proportional underestimation of stress. This highlights a major source of error in conventional analyzes, where elastic moduli, visco–elastic behavior, and passive force capacity may be prone to a systematic error. However, the consistency of our empirical data with a cylinder model suggests that – where such tracking is not feasible – a simple analytical correction based on the cylinder model may serve as a practical alternative (simulate the dynamically decreasing diameter). Importantly, this correction is now empirically validated, for the first time, and can help mitigate systematic underestimation in muscle biomechanics studies.

### 4.2 Continuous vs. Stepwise Stretching: Implications for Elastic Modulus Accuracy

By attributing fibre thinning during stretch, we challenged two commonly used protocols in biomechanics to assess axial fibre stiffness: continuous stress–strain vs. discrete stepwise “stretch‐jumps” (“stress‐relaxation” tests [29]). Although both approaches are often used interchangeably, our results revealed notable discrepancies in the computed restoration stress and the derived Young's modulus. Specifically, continuous stress–strain recordings at a commonly considered “quasi‐static” speed of 1 μm/s yielded consistently higher stress values and a steeper elastic slope compared to stepwise ‘stretch‐jumps’ followed by 1‐min relaxation periods. This suggests that, despite the relatively slow stretch rate (corresponding to 0.05% $L_{0}$/s for a 2 mm fibre), continuous protocols may still capture contributions from viscous or time‐dependent elastic components that are otherwise assumed to be in equilibrium for slow elongations. This residual component can be seen as a slow but steady force decline remaining at the beginning of the holding phase at 40% strain and confirming the still presence of viscous relaxation ([57] Figure 6A). This aligns with reports from other groups indicating that even modest stretch velocities as 0.05% $L_{0}$/s in rat EDL single fibres can elicit non‐negligible visco–elastic responses in passive stretches [27, 58], as well as in eccentric stretches [11].

Notably, the stress–strain‐derived Young's modulus in our study averaged ∼110 kPa – higher than the ∼86 kPa determined from stepwise ‘stretch‐jumps’, which likely better isolate the purely elastic plateau stress. These values are in agreement with previous reports, where Young's moduli for single fibres ranged between 20‐150  kPa (not accounting for diameter thinning) depending on fibre type, species, preparation (skinned vs. intact), and stretch protocol [24, 25, 59, 60, 61]. Whole muscle or fibre bundle preparations typically report larger values (>200 kPa, [24, 51, 62]) due to additional contributors to passive stiffness (e.g., connective tissue, extracellular matrix [63]), but also the highly ordered arrangement and interconnections of individual muscle fibres [50, 64].

A further comparison of stretch velocities supports the idea that viscous components are often included inadvertently: many groups describe their stretch rates in relative terms, commonly between 0.1%–1% $L_{0}$/s (e.g., [40, 65, 66]). Our protocol, though slower in absolute terms, is likely not exempted from rate‐dependent effects. Stretching even slower (∼nm/s) might reduce viscous contributions, but would impose impractically long recordings and technical challenges of super‐fine stepping motor or voice coil accuracies. Thus, for experiments aiming to isolate passive elastic restoration stress, stepwise “stretch‐jumps” paired with an adequate relaxation time present a technically more feasible and biologically robust alternative.

### 4.3 Stress–Strain Relationships Reveal a Calcium‐Dependent Modulation of Passive Stiffness

Our stress–strain recordings suggest that passive muscle fibre stiffness is modulated by calcium, even under conditions where active cross‐bridge cycling is chemically inhibited. Specifically, fibres exposed to high calcium (pCa 4.92) in the presence of BTS exhibited a significant increase in both restoration stress and Young's modulus compared to lower calcium conditions. Assuming that titin contributes substantially to this behavior, our findings are consistent with previous reports [13, 45, 67, 68, 69]. In these studies, two main mechanisms have been proposed to explain calcium‐dependent stiffening. The first attributes increased stiffness to a calcium‐dependent rise in titin's intrinsic spring stiffness, as calcium binds directly to specific regions, such as the PEVK or N2A segments. Reported stiffness increases range from ∼15% to ∼35%, depending on the preparation and experimental setup [67, 69]. The second mechanism suggests a reduction in titin–s free spring length through calcium‐facilitated titin–actin binding, effectively shifting titin's spring‐like properties toward shorter, stiffer working ranges [70, 71, 72, 73]. However, this interaction is believed to require access to actin binding sites, only available during effective cross‐bridge cycling. Indeed, Powers et al. (2014) [69] demonstrated that in rabbit *M. psoas* myofibrils treated with BDM (2,3‐Butanedione Monoxime) or after removal of troponin C, calcium could still enhance passive tension at long sarcomere lengths, even under cross‐bridge inhibition, likely through direct titin stiffening. However, this effect was absent within the physiological sarcomere length range, suggesting that calcium‐dependent titin–actin binding may require active cross‐bridge engagement to occur.

Given that BTS was present in our experiments to inhibit myosin II ATPase activity [74], active cross‐bridge cycling – and thus the structural engagement between actin and myosin – was effectively suppressed. While this leaves actin binding sites technically unoccupied, evidence suggests that titin–actin interactions require more than just availability of actin: they depend on prior or residual cross‐bridge activity to bring titin into close spatial proximity or to expose its actin‐binding domains [69]. Therefore, the calcium‐induced increase in passive stiffness observed in our stress–strain recordings under BTS treatment is unlikely to be primarily explained by titin‐actin coupling. Instead, our data are consistent with a mechanism of intrinsic titin stiffening via direct calcium binding to elastic domains such as the PEVK or N2A segments. Notably, Leonard et al. [75] reported comparable calcium‐dependent restoration forces in passively stretched myofibrils with and without cross‐bridge inhibition, further supporting the idea that titin alone can account for this stiffening effect in the absence of cross‐bridge cycling.

An additional consideration is the protocol‐specific nature of the observed stiffness increase. Our stress–strain recordings involved a continuous slow stretch at rather low speeds of 1 μm/s. While conventionally considered “quasi‐static” to isolate elastic properties, our direct comparison of stress–strain to “quasi‐instantaneous” stepwise “stretch‐jumps” has shown that even at this slow velocity, viscous components such as titin's PEVK domain may still contribute to measured stress ‐ particularly in calcium‐rich environments where titin–s mechanical response may shift toward a more viscous profile [46, 47, 48]. This may explain why a calcium‐dependent stiffening effect was detected in the stress–strain recordings but not in the stepwise‐stretch jumps, where viscous contributions were allowed to relax for 60 s while holding at a given stretch. In addition to the viscoelastic properties attributed to titin–s PEVK region, unfolding and refolding of immunoglobulin (Ig)‐like domains may also contribute to the observed protocol‐dependent mechanical behavior. Ig‐domain unfolding has been proposed as a potential contributor to passive elasticity under high‐force or sustained stretch conditions [47, 76, 77]. Continuous stretch protocols may promote progressive unfolding of titin Ig domains under sustained loading, whereas relaxation phases–as implemented in stepwise “stretch‐jump” protocols–can allow partial refolding and redistribution of strain within titin–s elastic elements [76, 78, 79]. As unfolding and refolding dynamics of Ig domains are force‐ and time‐dependent, differences in loading history between continuous and stepwise protocols may contribute to protocol‐dependent mechanical behavior [80]. This may contribute to differences in measured stiffness between the two protocols. However, given the moderate strain range and stress levels applied in the present study, as well as the relatively short duration of individual stretch phases, the contribution of Ig‐domain unfolding is likely secondary compared to viscoelastic effects associated with the PEVK region.

An additional layer of complexity arises when comparing passive restoration stress in calcium‐free conditions with and without myosin ATPase inhibition. In our study, fibres stretched at pCa 9.0 without BTS exhibited markedly higher restoration stress and Young's modulus (∼100 kPa ‐ consistent with cut single EDL fibre segments in Ref. [51]) than the same fibres under BTS treatment. This corroborates with previous reports suggesting that weak‐binding acto‐myosin interactions may persist in chemically skinned fibres even under relaxing conditions [81]. In this setting, slow continuous stretches may promote the recruitment or stabilization of weakly bound or pre‐power‐stroke cross‐bridges, which can contribute to increased stiffness during stretch [55, 82, 83]. These cross‐bridges, while not generating substantial isometric force, can resist elongation and, thereby, augment the measured restoration stress [83, 84]. BTS is designed to disrupt this interaction, however, its mechanism of action is dose‐dependent and may not entirely abolish all weak binding interactions at the molecular level [42]. The concentration used in our experiments (20 μM) is within the established effective range [42], but even so, complete elimination of acto‐myosin coupling cannot be guaranteed. Consequently, we cannot definitively resolve whether calcium‐dependent stiffening under BTS‐treated conditions is entirely independent of acto‐myosin interactions. Further studies, perhaps employing complementary inhibitors (e.g., non‐hydrolysable ATP analogues) will be required to unravel these overlapping mechanisms.

In summary, our observations in stress–strain recordings add to the growing body of evidence suggesting that titin functions as a dynamic, calcium‐sensitive spring in muscle fibres, and that the mechanical response of muscle is not merely dictated by acto‐myosin interactions but also by modulation of structural components in the sarcomere. However, the choice of biomechanical assessment protocol–continuous stretch vs. stepwise jumps–substantially impacts the ability to resolve these effects. This underscores the importance of integrating protocol design with mechanical interpretation, especially when investigating the complex interplay of viscoelastic and purely elastic elements in muscle biomechanics.

### 4.4 Dissecting Passive Biomechanics: How Stretch Protocols Shape the Interpretation of Calcium‐Dependent Muscle Stiffness

Our application of stepwise stretch‐jump protocols provided additional insights into the passive biomechanics of single muscle fibres under varying calcium conditions. In contrast to the findings from continuous stress–strain recordings, the stepwise approach did not reveal any significant differences in elastic stress or Young's modulus across calcium concentrations or BTS treatment. This discrepancy highlights a critical distinction between the two protocols and underscores the complex nature of passive muscle stiffness.

A plausible explanation for this observation may lie in the viscoelastic behavior of titin, particularly its PEVK/Ig region, which is known to contribute time‐dependent properties to muscle mechanics [46]. In continuous stress–strain recordings (1 μm/s or 0.05% $L_{0}$/s) both, elastic and viscous components contribute to stress (see Ref. [57] Figure 6A), especially in calcium‐rich environments where titin's conformation and stiffness may shift dynamically [46, 47]. In contrast, the stepwise stretch‐jump protocol includes prolonged holding phases at constant strain that allow for viscous elements to relax. The absence of calcium‐dependent changes in the stepwise data, therefore, suggests that the calcium sensitivity observed in continuous stretches may predominantly reflect modulations in titin–s viscous or time‐dependent properties.

An additional factor influencing this outcome is the potential contribution of weak‐binding cross‐bridges. In the control condition at pCa 9.0 without BTS, the Young's modulus derived from continuous stress–strain recordings was significantly higher than that obtained from stepwise “stretch‐jump” protocols. This may reflect that slow, continuous stretches (1 μm/s) prolong the lifetime of weak acto‐myosin interactions or increase their probability of formation, thereby contributing to the measured stiffness. In contrast, stretch‐jump protocols impose rapid length changes that are expected to transiently disrupt cross‐bridge attachments [75] and generate pronounced force peaks. In the present analysis, however, only the steady‐state plateau following a defined relaxation period was considered, thereby minimizing the direct contribution of cross‐bridge and viscous components to the quantified stiffness. The observation that calcium‐dependent stiffening was evident during continuous stretching but not under stepwise conditions, even within the same fibres, provides indirect but intriguing evidence that titin‐based stiffness modulation may be influenced by prior cross‐bridge activity, consistent with the concept of history‐dependent titin–actin interactions.

Yet, this conclusion must be interpreted with caution. Our evidence is indirect and protocol‐dependent, and alternative explanations such as remaining viscous elements, partial cross‐bridge inhibition by BTS, or calcium‐induced changes in other structural proteins cannot be entirely ruled out. Further research using targeted molecular approaches, titin–actin binding assays, or advanced molecular imaging techniques will be necessary to determine whether titin–s role as a calcium‐sensitive spring is strictly dependent on cross‐bridge formation, or if other regulatory mechanisms contribute to this behavior.

## 5 Conclusion

From a technical standpoint, our data emphasize the profound impact of experimental design on the interpretation of passive stiffness. Stretching at 1 μm/s (or 0.05% $L_{0}$/s), although commonly regarded as quasi‐static, may still probe viscous components–particularly within structural elements, potentially such as titin–s PEVK domain. Achieving truly elastic measurements would require to either significantly reduce stretch velocities (impractical in standard experimental workflows), or the use of stepwise protocols that explicitly allow for viscous relaxation. These considerations are essential for interpreting biomechanical data and for drawing conclusions about the molecular origins of stiffness modulation.

Beyond protocol selection, our findings highlight the critical importance of fibre‐wide structural analysis. Single muscle fibres present diameter variations along the longitudinal axis which can introduce substantial errors in stress computation if not accounted for. During stretch protocols, this issue becomes more pronounced, as fibres exhibit a measurable thinning of up to 13%–15% at 40% strain, resulting in a ∼27% underestimation of restoration stress when using static diameter assumptions. Therefore, tracking the same fibre segment during stretch by synchronising the FOV with the stretch controller is essential for accurate biomechanical assessments. When this is not feasible, applying an experimentally validated cylinder model to simulate diameter decrease provides a robust alternative, ensuring that stress calculations remain reliable.

In summary, the contrasting outcomes between stress–strain and stepwise “stretch‐jump” protocols reinforce the notion that passive muscle stiffness is not a monolithic property, but rather a dynamic interplay of elastic, viscous, and potentially weak‐binding elements. Calcium‐dependent modulation of this behavior appears to target both, elastic and visco–elastic components, with the balance between them dictated by the mechanical history and protocol used. These insights underscore the necessity of protocol‐aware interpretations and fibre‐wide structural assessment in muscle biomechanics research and highlight the *MyoRobot 3.0*–s capability to dissect these nuanced mechanical phenomena.

## Author Contributions

Conceptualization: M.H., and O.F.; Methodology: M.H., M.H. and M.T.; Software: M.H., M.H. and M.T.; Experiments: M.H., L.K., M.E.V. and M.T.; Formal Analysis: M.H., M.H. and M.T.; Data curation/visualization: M.H., M.H. and M.T.; Resources: L.K., M.E.V. and O.F.; Original draft preparation: M.H. and O.F. Reviewing and editing: O.F.; Supervision: M.H., and O.F.; Project administration: M.H., and O.F.; All authors have read and agreed to the published version of the manuscript.

## Funding

This research was internally funded.

## Conflicts of Interest

The authors O.F., M.H., and M.H. have filed a patent (DPMA) application regarding the motorized optics system employed within the *MyoRobot 3.0*.

## Data Availability

The data that support the findings of this study are available from the corresponding author upon reasonable request.

## Appendix A

### A.1 Mitigating Visual Artifacts in Real‐Time Fibre Diameter Tracking

While the integrated optical system of the *MyoRobot 3.0* enables continuous, frame‐accurate tracking of fibre diameter during mechanical testing, long‐term experiments inevitably expose the sample to visual artifacts. These can arise from ambient dust, residual debris from the mounted fibre, or remnants of the silk suture used for mechanical sample mounting. Such obstructions occasionally intersect with the fibre's optical contour, leading to transient irregularities or discontinuities in automated diameter detection.

To address this, we implemented a post‐processing algorithm capable of detecting and correcting such artifacts. The algorithm applies an adaptive z‐score‐based filtering strategy: local moving averages and standard deviations are computed in both forward and backward directions, and outliers are identified when diameter values fall outside the computed statistical thresholds. These outliers are replaced by linear interpolation, resulting in a smoothed, continuous diameter signal suitable for stress computation.

To evaluate the algorithm–s robustness, we first simulated artificial diameter traces overlaid with different types of disturbances: rectangular blocks, triangular ramps, and spike‐like interferences (Figure A1A–C, grey). In all cases, the algorithm successfully isolated and removed the artifacts, reconstructing the underlying diameter trajectory (black traces). Following this, we applied the same pipeline to real‐world diameter recordings from single muscle fibres (Figure A1D). Despite substantial and irregular artifacts in the raw data, the algorithm produced a reliable signal that preserved the general diameter trend during fibre stretch.

This filtering pipeline enables more robust stress computation under stretch. Specifically, it allows for normalization of force to dynamically adjust fibre cross‐sectional area – thus, reducing error margins caused by assuming a static diameter. Should real‐time image processing fail (e.g., due to noise), the experimentally supported cylinder model (Figure 4) can be applied as a fallback to simulate diameter changes. Either approach allows for correction of the assumption of constant fibre geometry under mechanical load.
